# Safety and adequacy of percutaneous kidney biopsy performed by nephrology trainees

**DOI:** 10.1186/s12882-017-0796-y

**Published:** 2018-01-15

**Authors:** Vittoria Esposito, Giulia Mazzon, Paola Baiardi, Massimo Torreggiani, Luca Semeraro, Davide Catucci, Marco Colucci, Alice Mariotto, Fabrizio Grosjean, Giacomo Bovio, Ciro Esposito

**Affiliations:** 1 Unit of Nephrology and Dialysis, ICS Maugeri, Via S. Maugeri 10, 27100 Pavia, Italy; 2 Unit of Nephrology and Dialysis, ICS Maugeri, University of Pavia, Via S. Maugeri 10, 27100 Pavia, Italy; 3 Science Department, ICS Maugeri, Pavia, Italy; 4 Unit of Nephrology, Policlinico San Matteo, Pavia, Italy; 5 Unit of Palliative Care, ICS Maugeri, Pavia, Italy

**Keywords:** Kidney biopsy, Trainee, Complications, Risk factors

## Abstract

**Background:**

Recently there has been a progressive loss of specialty related skills for nephrologists. Among the skills we find the kidney biopsy that has a central role in diagnosis of renal parenchymal disease. One of the causes might be the belief that the kidney biopsy should be performed only in larger Centers which can rely on the presence of a renal pathologist and on nephrologists with a large experience. This trend may increase in the short term procedural safety but may limit the chance of in training nephrologists to become confident with the technique.

**Methods:**

We evaluated renal biopsies performed from May 2002 to October 2016 in our Hospital, a mid-sized facility to determine whether the occurrence of complications would be comparable to those reported in literature and whether the increase in the number of biopsy performing physicians including nephrology fellows which took place since January 2012, after our Nephrology Unit became academic, would be associated to an increase of complications or a reduction of diagnostic power of renal biopsies. Three hundred thirty seven biopsies were evaluated. Patients underwent ultrasound guided percutaneous renal biopsy using a 14 G core needle loaded on a biopsy gun. Observation lasted for 24 h, we evaluated hemoglobin levels 6 and 24 h and kidney ultrasound 24 h after the biopsy.

**Results:**

Complications occurred in 18.7% of patients, of these only 1,2% were major complications. Complications were more common in female (28%) compared to male patients (14,8%) (*p* = 0.004). We found no correlation between diagnosis, kidney function and complication rates; hypertension was not associated to a higher risk in complications. The increase of biopsy performing personnel was not associated to an increase in complication rates (18,7% both pre and post 2012) or with an increase of major complications (1.2% vs 1,2%).

**Conclusions:**

Kidney biopsy can be safely performed in mid-sized hospitals. Safety and adequacy are guaranteed even if the procedure is performed by a larger number of less experienced nephrologists as long as under tutor supervision, thus kidney biopsy should become an integral part of a nephrology fellow training allowing more widespread diffusion of this technique.

## Background

Nephrology is a specialty in need of resuscitation according to a forum published on Kidney International in 2009 [1]. The forum deals with the reported workforce shortage of nephrologists in some developed countries. Italy is also facing a similar situation since according to a recent census carried out by the Italian Society of Nephrology (SIN), there are almost 3 million patients with CKD and only 2600 nephrologists. In 2005 a questionnaire sent to all physician trainees eligible to sit the clinical component of the Royal Australian College of Physicians examination revealed that one of the main barriers cited among the reasons nephrology was excluded as a future career was a negative impression of the career developed during a nephrology rotation. Many of the trainees found the topic to be unappealing [2]. According to a survey from 5 medical schools in the United States: renal pathophysiology courses were too complex, lacked relevance or simply failed to stimulate interest [3]. Many students reported minimal nephrology exposure during their clinical rotations and even fewer were aware of the procedural work in nephrology, a feature than often attracts students to other specialties. Also among nephrology fellows, reasons for dissatisfaction with their career choice included overall poor experience during fellowship training and the close association with general internal medicine [4]. Actually over the last decades there has been a progressive loss of specialty related skills for nephrologists determining a dramatic change in the average nephrologist’s job description. Arterio-venous access for extracorporeal dialysis are performed more and more often by vascular surgeons, catheters for dialysis are placed by critical care physicians. Finally in recent times percutaneous renal biopsies have been taken over by non-nephrologists particularly radiologists in many institutions. The kidney biopsy which represents a central diagnostic tool for renal parenchymal disease is done by radiologist in more than 40% of cases in United States [5] and in Korea only 26.1% of renal biopsies are performed by nephrologists and 42.9% by radiologists [6]. The reasons behind the progressive abandonment of the kidney biopsy are many, the main being the progressive trend to centralize the bioptic procedure in larger hospital facilities in order to guarantee optimal technical expertise of the biopsy performing nephrologist and pathologist, reducing the risk of biopsy related complications and increasing diagnostic power of the procedure. Although the idea of improving quality and safety of a procedure may seem optimal theoretically, it has led to several problems. Nephrology fellows often complete their training without having the chance of learning how to perform a renal biopsy and in some cases never even have a chance of watching how the procedure is performed. As mentioned above this has been considered among the factors which have led to a progressive loss in interest towards nephrology for young doctors.

In this single center retrospective observational study we evaluated patients undergoing renal biopsy from May 2002 to October 2016. During this time frame our Nephrology Unit in ICS Maugeri SpA became academic (January 2012) thus acquiring nephrology fellows which were actively involved in clinical practice including renal biopsy performance. Aim of our study was evaluating whether the increase in number of biopsy-performing physicians including nephrology fellows from January 2012 would be associated to an increase of complications or a reduction of diagnostic power of renal biopsies and to determine whether the complication rate in our average sized hospital would be comparable to rates reported in literature by larger hospitals.

## Methods

This study evaluated kidney biopsies performed at ICS Maugeri from May 2002 to October 2016. Our cohort included 337 patients, all of which had consented to the biopsy procedure. Only adult patients were included whereas pediatric patients and patients undergoing a biopsy as a diagnostic procedure in the presence of a solid renal mass were excluded.

For all the included patients the following data, available in medical charts, were collected: age, gender, medical history, concomitant medications, bio-humoral testing such as serum creatinine, hemoglobin, platelet count, proteinuria, hematuria, needle size, number of glomeruli including sclerotic glomeruli per biopsy specimen, immunofluorescence report, number of passes performed, microscopic observation of freshly acquired specimen, diagnosis, presence of complications including complication severity.

Complications were classified as follows.

Minor complications: arteriovenous fistula, minor hematoma spontaneously reabsorbed, gross hematuria, post-procedural hypotension.

Major complications: need of blood transfusions, hematoma requiring drainage.

All patients needing to perform a kidney biopsy were admitted the day prior to the procedure in order to perform preliminary exams. Patients known to be on anticoagulant or antiplatelet therapy were contacted in order to stop medication at least 1 week prior the procedure switching to heparin as needed.

Routine laboratory tests performed on admission included complete blood count, coagulation testing, biochemistry (including creatinine, urea and electrolytes, urinalysis and 24 h proteinuria and evaluation of autoimmunity and complement count). On admission all patients also underwent a renal ultrasound in order to confirm the presence of adequate sized kidneys excluding the existence of anatomical abnormalities which may represent contraindication to the procedure.

Biopsies were performed 24 h after admission in the early morning.

All patients underwent real time-ultrasound guided percutaneous renal biopsy according to local practice. Briefly patients were placed in the prone position with a pillow under the abdomen in order to reduce lumbar lordosis (transplanted patients were placed supine); blood pressure and heart rate where checked before the procedure and pressure cuff was left for subsequent measures during the procedure. Before starting a peripheral vein was cannulated to guarantee a rapid infusion line in case of intra-procedural hypotension. Two operators, an assistant and a more experienced nephrologist acting as supervisor were always present. The lower pole of the left kidney was located by ultrasound subsequently the skin was disinfected with 10% povidone iodine, prior to injecting local anesthesia with 1% lidocaine. A small incision was made to facilitate introduction of the biopsy needle. All biopsies were performed using a Bard automated biopsy gun loaded with a 14 Gauge tru-cut needle. Under real time-ultrasound guidance the needle was advanced by the second operator until reaching the lower pole of the kidney and subsequently fired and removed.

Until 2012 each core was only observed macroscopically for size, after January 2102 observation was systematically performed under a stereomicroscope with 20X magnification to verify the presence of glomeruli in order to avoid other passes if not needed. After the procedure a renal ultrasound was systematically performed to evaluate the presence of hematomas. A flat dressing and ice were applied on the area. After the biopsy, patients were asked to stay in prone position for the next 2 h and kept bed bound until the next morning. Every half hour blood pressure and heart rate were monitored and the first void was checked for hematuria. Complete blood count was performed 6 h after biopsy and repeated at 24 h from procedure. A ultrasound control was performed in every patient 24 h after procedure. If no complications occurred patients were discharged the day after the procedure and afterward contacted for results.

Descriptive statistics were performed for all the collected variables. Frequencies and percentages or means and standard deviations were reported for qualitative or quantitative variables, respectively. Ordinal variables were summarized as median, minimum and maximum of the distributions. Statistical associations between factors potentially influencing the occurrence of biopsy-related complications and complications were tested by means of chi-square test. Mann-Whitney U test was used to assess differences in the median number of passes needed to grant adequate diagnostic power of the biopsy before and after 2012. A statistical significance level of 0.05 was chosen. SPSS Statistical Software was used for the analyses.

## Results

Three hundred thirty seven patients undergoing a kidney biopsy in our Unit between May 2002 and October 2016 were included in the study. 50.7% (171 patients) underwent a renal biopsy between May 2002 and December 2011 and 49.3% (166 patients) form January 2012 to October 2016 (Table 1).

**Table 1:** Kidney biopsy complications risk factors. Univariate analysis

	Complications	*p* value
Gender		
Male	35 (15%)	
Female	28 (28%)	0.004
AHD		
None	23 (29%)	
< 3	35 (16%)	
≥ 3	5 (14%)	0.025
sCr		
< 2	41 (18%)	
≥ 2	22 (20%)	0.588
AA		
No	60 (18%)	
Yes	3 (25%)	0.568
AID		
No	4 (13%)	
Yes	57 (19%)	0.380
VD		
No	20 (17%)	
Yes	41 (20%)	0.599
ND		
No	8 (22%)	
Yes	53 (18%)	0.574
Primary GD		
No	27 (18%)	
Yes	34 (19%)	0.742

Risk factors for postbiopsy complications in kidney biopsies performed at the Unit of Nephology and Dialysis, ICS Maugeri, Pavia, Italy between 2002 and 2016. All major risk factors were evaluated by univariate analysis. The number of complications for each risk factor are indicated. In the bracket the corresponding percentage is indicated. (*p* < 0.05 was considered significant). (*AHD* antihypertensive drug, *sCr* serum creatinine, *AA* amyloidosis, *AID* autoimmune disease, *VD* vascular disease, *ND* diagnosis of neoplasia, *GD* glomerular disease)

Our cohort consisted of 100 females (29.7%) and 237 males (70.3%); mean age at the time of biopsy was 58.0 ± 15.8 years. Forty seven patients (13.9%) were positive for hepatitis C virus (HCV). Hypertension occurred in 70.6% of patients; among hypertensive patients 10.4% were defined as having a severe hypertension based on the need to take more than three antihypertensive agents.

Complications occurred in 18.7% of patients with major complications occurring in 1.2% of patients. Interestingly complication rates was comparable when considering the interval before and after 2012. Also complication rate and severity were comparable when considering the interval before and after January 2012 *p* = 0.993 (Table 2).

**Table 2:** Biopsy complications and specimen adequacy before and after 2012

	2002–2011 *n* = 171	2012–2016 *n* = 166	*p* value
Complications			
	32 (19%)	31 (19%)	0.993
Diagnostic Biopsies			
	164 (96%)	164 (99%)	0.170

Biopsy complications and adequacy of the specimen in kidney biopsies performed at the Unit of Nephrology and Dialysis, ICS Maugeri, Pavia, Italy. Biopsies performed before and after 2012 are compared. The number of complications and of diagnostic biopsies before and after 2012 are indicated. In the bracket the corresponding percentage is indicated. (*p* < 0.05 is considered significant)

The diagnostic power of the bioptic procedure was not reduced starting form 2012. Our results show a trend towards a reduction of non diagnostic biopsies, 7 (4.1%) vs 2 (1.2%) before and after 2012 respectively, associated to a simultaneous statistically significant reduction of the median number of passes required to gain an adequate core (*p* < 0.001). Based on previously published data [7] we analyzed in our population the impact on complication rate of the following factors: sex, arterial hypertension and its severity, severity of renal function impairment, activation of immune system, diagnosis of oncologic diseases or amyloidosis, diagnosis of primary or secondary glomerular disease (Table 1).

The results of our study show that the incidence of complications, both minor and major was significantly higher in females (28%) compared to males (15%) (*p* < 0.005) thus confirming the observation that female patients are at higher risk of developing procedural associated complications.

Two hundred fifty eight patients were on antihypertensive treatment and 35 were classified as having severe hypertension based on the need to take more than three antihypertensive agents to achieve adequate blood pressure control. Interestingly complications were more common in normotensive compared to hypertensive patients (*p* < 0.05). Also the severity of hypertension had no correlation with the complication rate.

To evaluate the existence of a possible correlation between kidney function and complication rate we next divided our cohort based on creatinine value stratifying patients with creatinine values below 2 mg/dl and above 2 mg/dl. Our data show that complications were not increased in patients with more severe renal function impairment (*p* = 0.588). Our population included 9 cases of amyloidosis. Although the diagnosis of amyloidosis has been suggested to be a predisposing factor for bleeding due to acquired coagulation factor deficiencies and to increased stiffness of vessels due to amyloid deposition we were not able to demonstrate an increase in biopsy related complications in this population compared to control group. No increase in complication incidence was found in patients with amyloidosis, activation of the immune system, oncologic conditions or vascular diseases nor did we find any difference in complication rate based on the presence of a primitive or secondary glomerular disorder.

## Discussion

Kidney biopsy is a very important diagnostic tool for nephrologists and has always been considered a characteristic of nephrologist’s job description. Recently however kidney biopsy has been increasingly reserved to larger hospitals with an experienced nephrologist and pathologist to guarantee maximum procedural safety and diagnostic power or it has been taken over by other specialists. The result is a reduced appeal of Nephrology specialty for young trainees. Eventually young nephrologists miss the technical expertise to perform a renal biopsy due to lack of training. In line with the guidelines given by the American Society of Nephrology our middle sized academic hospital has since 2012 promoted the training of young nephrologists even in biopsy performing.

In our study we evaluated if procedural safety and diagnostic power of the renal biopsy in a middle sized hospital could be comparable to those reported by larger hospitals also assessing procedural safety and adequacy of biopies performed by less experienced operators, including fellows, under tutor supervision.

Our data showed minor complications in 17.3% of biopsies and major complications in 1.2% of cases. This incidence rate was in line with the one reported by larger centers especially if we consider that our definition of minor complications included millimetric sized hematomas which are very often considered an unavoidable consequence of the bioptic procedure just as much as microscopic hematuria [8]. Furthermore all the biopsies of our study were performed using a 14-gauge needle that has been shown to be associated with a higher rate of complications compared to the most used 16 and 18 gauge needles [9–12]. Manno and colleagues found no differences in complications rate between 14 and 16 gauge needles in their series of biopsies [13]. However they evaluated biopsies performed in patients with low bleeding risk and this could have blunted the differences between the two needles. It may be difficult to compare differences in complications rates among studies because they can vary substantially due to confounding issues such as the type of the study (retrospective vs. prospective), patient mix, the ultrasound machine used, the needle type or gauge used, and of course the operator performing the biopsy. However one of the largest (1055 patients), prospective, single-center study with PRB performed in adults at an academic institution using real-time ultrasound and 14-gauge needles showed results that were comparable to the results of our study [14]. Furthermore in the study by Korbert and colleagues the rate of complications was similar and the majority of procedures were performed by nephrology fellows. In our cohort there were no cases of death or nephrectomy and although we described one arteriovenous fistula it did not require any treatment. As reported by several studies [13, 15] in our cohort female patients were at higher risk of procedural related complications. The increased bleeding risk observed in women has been explained with their different body composition. Women in fact have a greater percentage of fat mass that could increase the bleeding into perirenal tissues [13]. The role of hypertension as a risk factor for bleeding after a kidney biopsy is still controversial, our study seems to confirm that hypertension is not a major risk factor for postbiopsy complications as reported by previous studies [8, 15]. Surprisingly postbiopsy complications were more common in the group of patients with normal blood pressure. To note, however, patients in our study were defined as hypertensive based on the need for antihypertensive drugs and moreover all hypertensive patients were treated in order to achieve blood pressure < 140/90 mmHg before the procedure and this could have affected the results reducing the risk of postbiopsy bleeding in hypertensive patients. Our results show that an adequately controlled hypertension, although severe, does not represent a contraindication to the bioptic procedure. We were unable to find any other clinical conditions increasing the incidence of procedural risk such as amyloidosis or advanced renal failure as reported by some studies [16, 17]. All together our data seems to show that the biopsy procedure can be performed safely in middle sized hospitals as the incidence of complications in our center is equal to the one described in literature. Interestingly, complication rate in our center was equal in the timespan before and after 2012. Before 2012 only one very skilled nephrologist performed kidney biopsies whereas after 2012 several nephrologists, mostly young trainees performed the procedure during their turnation. This very important data suggests that an increase in the number of biopsy performing personnel including nephrology fellows as long as under tutor guide does not imply a reduction of procedural safety. Based on this, the training requirement (ability to independently perform percutaneous kidney biopsy of both native and transplanted kidneys) proposed by the American Board of Internal Medicine (ABIM) and the Accreditation Council for Graduate Medical Education (ACGME) for nephrology fellows appears feasable [18]. However, although the Board of Internal Medicine requires that nephrology fellows be trained to perform percutaneous kidney biopsy, the Renal Pathology Society and some authors underline that a kidney biopsy should only be done by someone skillful in performing the procedure [19, 20]. Dawoud and colleagues even proposed a simulation tool that mimics biopsy conditions in human patients to improve confidence and procedural skill competence of trainees [21]. In their study individual fellows practiced repeatedly ultrasound-guided renal biopsy procedures using a porcine kidney/turkey breast phantom until they attained reasonable accuracy and gained confidence. They were guided by an experienced operator. The effect of this simulation training on trainees’ procedural competence was evaluated by comparing outcomes of renal biopsies performed by fellows before and after the implementation of the simulation training. The study showed that the implementation of the simulation training reduced the severity of biopsy-associated bleeding complications. The use of simulation tools could be implemented in the curriculum of nephrology fellows to improve the accuracy and confidence of nephrology fellows not only for renal biopsy procedure but also for arterio-venous fistula and central catheter placement. Our results confirm those obtained by Chung and colleagues [22] who evaluated the safety of kidney biopsy according to practitioner and ultrasound technique. In their study the authors compared complication rate of kidney biopsy performed by nephrologists and radiologists. Nephrologists in their study were first and second year fellows. Their results show that percutaneous renal biopsy performed by young trainees was not inferior to that performed by expert ultrasound radiologists. Furthermore the mean number of glomeruli in renal tissue obtained by nephrologists was significantly higher than that obtained by radiologists. In our study the number of adequate biopsies was similar before and after 2012 but the number of passes was significantly lower after 2012 demonstrating, as shown in the study by Chung, that young nephrology trainees can obtain a better biopsy core. To date, however, our study is the first one comparing kidney biopsies performed by a skilled nephrologist to those performed by unskilled nephrologists and trainees. Furthermore according to a study by Corapi and coworkers [8] our cohort of patients biopsied after 2012 cannot be considered low risk patients since mean age was quite high (59.24 ± 15.6 years) much more than any previous study, a large percentage of patients had a reduced renal function (creatinine >2 mg/dl, 36.7% of patients) and many presented with acute kidney failure. Another essential data of our study is the one concerning the diagnostic power of renal biopsy. Our study points out adequacy of procedural diagnostic power in our Hospital. Of the 337 biopsies evaluated in fact, 97.3% proved adequate, allowing diagnosis, whereas only 9 did not allow diagnosis. Interestingly we show that starting from 2012 the number of non diagnostic biopsies decreased from 7 (4.1%) to 2 (1.2%). Although not statistically significant this data, which is probably secondary to the practice of observing the freshly obtained sample by light microscopy to evaluate adequacy of the bioptic specimen, once more shows that diagnostic power of the bioptic procedure does not change if it is performed by less expert operators. In the present paper we focused on the ability of young fellows to perform kidney biopsies under the guidance of an experienced nephrologist. However to perform kidney biopsy is only part of the task. Glomerular diseases are rare and it is mandatory, in order to make a diagnosis, to have the biopsy evaluated by an experience pathologist who can describe the biopsy as soon as possible to guide the treatment.

We acknowledge that the present study has many limitations. First of all it is a retrospective study and the biopsies considered were performed in two rather long periods of time. We did not find a significant dfference in outcomes however many variables could have affected outcomes such as different patients characteristics, different technical approach and different quality of ultrasound machines. Patients undergoing kidney biopsy in the two study periods had similar characteristics and no differences were found in kidney diseases. We used the same ultrasound machine throughout the period examined, a machine that was only older when used by young trainees. The only remarkable difference in the techique used to perform the kidney biopsy was the analysis of the tissue by light microscopy to check for glomeruli.

Since a16G needle is perfectly adequate for obtaining good histology sample it could be used when a more inexperienced operator is performing the procedure, increasing the safety of the procedure.

## Conclusion

In conclusion performing kidney biopsies in middle sized Hospitals appears safe and garantees good diagnostic power. The increase in number in biopsy-performing personnel, including unexperienced nephrology fellows, does not increase complication rate or severity and is associated to comparable diagnostic power. Thus the kidney biopsy should be an integral part of nephrology fellows training allowing a more widespread diffusion of the technique to smaller and more peripheral centers.

## Abbreviations

AA: Amyloidosis
ABIM: American Board of Internal Medicine
ACGME: Accreditation Council for Graduate Medical Education
AHD: Antihypertensive drugs
AID: Autoimmune disease
CKD: Chronic Kidney Disease
GD: Glomerular disease
ND: Diagnosis of neoplasia
sCr: Serum creatinine
SIN: Società Italiana di Nefrologia
SPSS: Statistical Package for Social Science
VD: Vascular disease
